# User engagement with organizational mHealth stress management intervention – A mixed methods study

**DOI:** 10.1016/j.invent.2023.100704

**Published:** 2024-01-02

**Authors:** Leo Kowalski, Anna Finnes, Sabine Koch, Aleksandra Bujacz

**Affiliations:** a Department of Learning, Informatics, Management and Ethics, Health Informatics Centre, Karolinska Institutet, Tomtebodavägen 18a, 171 65 Solna, Stockholm, Sweden; b Department of Clinical Neuroscience, Division of Psychology, Karolinska Institutet, Nobels Väg 9, 171 65 Solna, Stockholm, Sweden; c Academic Primary Healthcare Centre, Region Stockholm, Solnavägen 1E, 113 65, Stockholm, Sweden

**Keywords:** mHealth, Organizational intervention, User engagement, Mixed methods

## Abstract

Mobile health (mHealth) demonstrates great promise for providing effective and accessible interventions within an organizational context. Compared with traditional workplace interventions, mHealth solutions may be significantly more scalable and easier to standardize. However, inadequate user engagement is a major challenge with mHealth solutions that can negatively impact the potential benefits of an intervention. More research is needed to better understand how to ensure sufficient engagement, which is essential for designing and implementing effective interventions. To address this issue, this study employed a mixed methods approach to investigate what factors influence user engagement with an organizational mHealth intervention. Quantitative data were collected using surveys (n = 1267), and semi-structured interviews were conducted with a subset of participants (n = 17). Primary findings indicate that short and consistent interactions as well as user intention are key drivers of engagement. These results may inform future development of interventions to increase engagement and effectiveness.

## Highlights

• App engagement rating is linked with adherence to mHealth intervention.
• Perceived effort and user intention are key drivers of engagement.
• Short and consistent interactions may support habit formation and so increase engagement.
• Social workplace factors may be important for encouraging engagement.

## 1 Introduction

### 1.1 Background and motivation

Organizational interventions can be defined as structured programs aiming to effect positive change within an organization. While these may target different aspects of work, interventions promoting health are especially important for supporting the health and well-being of employees. Such initiatives may provide benefits for the overall organization as well, for instance by mitigating health problems associated with long-term sick-leave, turnover, and overall absenteeism (Estevez Cores et al., 2021; Hassard et al., 2018). As a corollary, successful interventions have been shown to positively impact job satisfaction, work engagement, and productivity, contributing to the overall benefit of both employees and the organization (Knight et al., 2017).

While organizational interventions have proven effective in many instances, they have several limitations with regard to implementation (Nielsen and Randall, 2015). Traditional workplace interventions – such as workshops, coaching, and counseling – are notoriously challenging to standardize and scale. Since on-site interventions must fit within the unique organizational structure of a workplace they have to be tailored for each individual organization, making it very difficult to develop a standardized solution that can easily be implemented in different workplaces (von Thiele Schwarz et al., 2021).

The use of mobile health (mHealth) – mobile technology designed to support health - may aid in addressing these challenges, showing great potential as effective alternatives for implementing interventions within an organizational context (Howarth et al., 2018; Phillips et al., 2019). Compared with on-site workplace interventions mHealth solutions are significantly easier to scale and standardize - a standardized intervention can be made available on a large scale without having to make major changes in either the organization or the intervention. The use of mHealth solutions may also increase accessibility and lower the threshold for employees to seek help, for instance by being available at the users´ convenience as well as reducing potential stigma associated with attending in-person interventions (Torous et al., 2021; Ebert et al., 2014).

Mobile phones are increasingly used as tools to improve health and well-being, providing unique possibilities for improving healthcare practice and research (Marzano et al., 2015; Price et al., 2014). mHealth interventions for treating health problems show positive effects on a wide range of health outcomes - including anxiety, depression, and physical activity (Schueller et al., 2017; Mönninghoff et al., 2021). Given the prevalence of mobile phones in today's world, mHealth opens up novel opportunities for making organizational health interventions accessible on a wide scale.

Though mHealth solutions provide preliminary hope for implementing accessible, effective, and scalable interventions in the workplace, a major challenge with digital interventions is user engagement – the extent to which users start using and repeatedly engage with a particular technology (Meyerowitz-Katz et al., 2020; Borghouts et al., 2021). Many studies report high dropout rates and low protocol adherence, an issue common enough to be known as *the law of attrition*, “the observation that in any eHealth trial a substantial proportion of users drop out before completion or stop using the application” (Eysenbach, 2005).

Insufficient user engagement is problematic because it may negatively impact the potential effectiveness of an intervention – a certain degree of engagement is necessary for an intervention to promote positive health outcomes (Yardley et al., 2016). Ratings on the App Engagement Scale, for instance, have been shown to mediate the effectiveness of a mental health application, while adherence rates are closely linked with intervention outcomes (Bakker and Rickard, 2018; Donkin et al., 2011; Yang et al., 2022). Ensuring sufficient engagement is thus a crucial part of mHealth design, and the failure to do so results in less effective interventions.

User engagement is a multi-faceted phenomenon which may refer both to the *amount* people use an application, as well as the attentive and immersive *quality* of being focused on the technology (Perski et al., 2017). As such, both quantitative and qualitative measures of engagement are frequently reported in the literature. Common quantitative measures of engagement include *uptake*, the degree to which people in the target population start using an intervention, and *adherence*, the degree to which users follow the intended intervention protocol (Borghouts et al., 2021). Both uptake and adherence are critical aspects of engagement since it is important both to motivate people to start using an intervention as well as sustaining engagement throughout the intervention to receive optimal benefits.

Given the importance of these constructs for an effective intervention, it is necessary to understand the underlying reasons which influence uptake and adherence. Several factors have been identified which affect engagement with digital tools, including perceived benefit, ease of use, reminders, tailoring, and many others (Jakob et al., 2022; Marangunić and Granić, 2015). A useful framework for conceptualizing the adoption and utilization of technology in an organizational context is the UTAUT model – Unified Theory of Acceptance and Use of Technology (Venkatesh et al., 2003). UTAUT was developed as a synthesis of several models of technology acceptance and has since been widely used (Tamilmani et al., 2021).

Employing the UTAUT framework within a qualitative approach may be a fruitful way of reaching a deeper understanding of the reasons behind user engagement (Bixter et al., 2019). Qualitative data are commonly used within interaction design and mHealth research to better understand the user experience during development (Molina-Recio et al., 2020). This kind of data in combination with quantitative measures – as in mixed methods studies - may lead to novel insights regarding the factors which influence uptake and adherence with mHealth interventions (Zhang et al., 2019). These insights are critical for designing engaging interventions which promote optimal health benefits.

Given the widespread problem of inadequate user engagement and the potential of using digital interventions within an organizational context, it is of great interest to elucidate how to engage intervention users. Within an organizational context, there is a lack of knowledge regarding how to effectively implement mHealth solutions to successfully engage employees. More research is urgently needed to understand the factors which promote engagement and thus improving effectiveness of mHealth interventions in the workplace.

### 1.2 Aim

Current knowledge and practice around organizational mHealth interventions is inadequate to ensure sufficient engagement among employees. The aim of the study is to gain a deeper understanding of the factors promoting engagement, i.e., uptake and adherence, with organizational mHealth interventions. This knowledge may be used to inform design principles and implementation strategies promoting engagement.

The two primary research questions regard the *uptake* and *adherence* of mHealth interventions.

1) Uptake – What predicts and explains whether a participant registers in the application or not?
2) Adherence – What predicts and explains how often a participant will use the application?

## 2 Methods

### 2.1 Study design

**Fig. 1:**
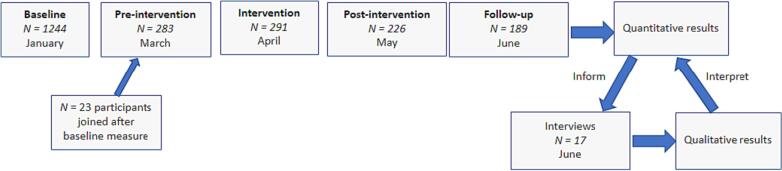
Flowchart of the data collection process.

This study employs a mixed methods sequential explanatory design (Ivankova et al., 2006). Mixed methods research uses both quantitative and qualitative data with the aim of integrating the results to gain insights that would not be possible by analyzing the data types separately. Data were collected in sequence at separate time-points, initially collecting quantitative data followed by a qualitative data collection as shown in Fig. 1.

Quantitative data are used to identify factors which predict engagement while qualitative data are used to *explain* in what way these factors influence engagement. For instance, a quantitative analysis may indicate that a particular measure predicts higher engagement, but qualitative data are necessary to interpret how and why the measure increases user engagement. Table 2 shows how quantitative and qualitative methods have been integrated.

#### 2.1.1 Quantitative

The quantitative data collection included four surveys - baseline, pre-intervention, post-intervention, and follow-up - over the course of six months. Participants also completed a month-long mHealth intervention between the pre-intervention and post-intervention measures. The full data collection was part of a larger experimental study, however, for the purposes of this study, only baseline and pre-intervention measures are included since these are used to predict engagement. Data from the other measures will be presented elsewhere.

Baseline measure was conducted via an online survey tool (Artologik, 2021). This survey included questionnaires measuring mental health (e.g., burnout and anxiety as referenced in Section 2.4.2) as well as single-item questions regarding the working environment (e.g., “During the last week, how often have you worked with Covid-19 patients?”). In the survey, participants could choose to take part in a month-long mHealth intervention administered through a mobile application. All subsequent data collection was conducted through the mobile application.

#### 2.1.2 Qualitative

Qualitative data consisted of semi-structured interviews conducted by the first author of the paper (Kallio et al., 2016). Interviews were conducted over Zoom or phone and audio was recorded. All interviews were transcribed verbatim. The interview guide is available in an Appendix.

### 2.2 mHealth intervention

The study evaluated user engagement in the context of a month-long mHealth stress management intervention developed by the research team (Bujacz and Kowalski, 2022). During the course of a month, the intervention prompts users daily to self-monitor their mood as well as providing information regarding stress management and recovery. Each evening, a notification reminds users to open the application and complete the intervention. Each daily interaction is intended to be very easy and quick, not taking up more than a few minutes of time.

Swedish healthcare workers were invited via e-mail provided by their employers to take part in a study of work-related stress reactions during the Covid-19 pandemic. Employees were initially informed about the study by their managers, and subsequently received an email from the research team. Healthcare workers (N = 1267) agreed to take part in the study during December 2020 – June 2021. Ethical approval was granted by the Swedish Ethical Review Authority (reference numbers 2020–01795 and 2022–01546-02). Table 1 shows demographic characteristics of all participants.

After the follow-up measure, all 1287 participants were invited to take part in an interview regarding their experience of using the intervention. A total of 58 participants agreed to an interview of which 17 were included in the qualitative sample. These participants were divided into three groups based on uptake and adherence metrics: (1) a high-adherence group (N = 7) who completed at least 14 out of 28 days of the intervention, (2) a low-adherence group (N = 5) who completed <14 days of the intervention, and (3) a no-uptake group (N = 5) who chose not to start the intervention at all.

Inclusion was based on having balanced amounts of participants in each group and, to the extent possible, sampling participants based on demographic characteristics such as age, gender, type of work, and work-experience in order to have a diverse respondent group. Due to difficulties recruiting participants to the no-uptake group and aiming for equal group sizes, subsets of participants were interviewed sequentially. A final sample size of 17 was decided upon as saturation was reached during analysis at this point.

### 2.3 Participants

**Table 1:** Descriptive statistics from all samples.

Group	Baseline survey (n = 1244)	Pre-intervention (n = 283)	Qualitative sample (n = 17)
Age, mean (sd)	46.1 (12.2)	45.4 (12.1)	43.3 (13.1)
Gender, n (%) Female Male	964 (78) 270 (12)	230 (82.4) 49 (17.6)	10 (58.8) 7 (41.2)
Occupation, n (%) Assistant nurse Nurse Physician Admin staff Other occupations	187 (15.3) 363 (29.7) 190 (15.5) 158 (12.9) 326 (26.6)	16 (5.7) 74 (26.4) 46 (16.4) 50 (17.9) 94 (33.6)	0 6 (35.3) 3 (17.6) 1 (5.9) 7 (41.1)
Work experience, n (%) 0–5 years 6–10 years 11+ years	302 (25.4) 204 (17.2) 681 (57.4)	70 (25.9) 47 (17.4) 153 (56.7)	4 (25) 4 (25) 8 (50)
Mental health measures, mean (sd) Burnout Depression Ptsd Anxiety Stress	2.33 (0.72) 1.73 (0.82) 2.03 (0.98) 1.63 (0.65) 2.44 (0.71)	2.42 (0.61) 1.72 (0.74) 2.06 (0.93) 1.67 (0.61) 2.46 (0.69)	2.52 (0.71) 1.76 (0.75) 2.20 (1.03) 1.82 (0.55) 2.54 (0.63)

*Note.* Values in baseline survey and qualitative sample are taken from baseline measure. Values from pre-intervention survey are taken from pre-intervention measure.

**Table 2:** Implementation matrix showing all data collection methods, analysis, and sample for each research question.

	Quantitative	Qualitative
Uptake		
*Research question:*	What predicts whether a participant will choose to register in the mobile application?	What explains why participants did or did not choose to register in the app?
*Sample:*	1244 participants in baseline survey.	17 participants who agreed and were chosen to participate in an interview.
*Data collection:*	Baseline survey collecting data on demographics and mental health measures.	Semi-structured interviews including uptake and opt-out participants.
*Data analysis:*	Binomial logistic regression.	Thematic analysis. Comparing themes between uptake and no-uptake groups.
Adherence		
*Research question:*	What predicts how often participants use the application?	What explains why participants used the application to the extent they did?
*Sample:*	283 participants who completed the pre-intervention measure.	17 participants who agreed and were chosen to participate in an interview.
*Data collection:*	Pre-intervention measure collecting data on demographics and mental health measures.	Semi-structured interviews including high-adherence and low-adherence participants.
*Data analysis:*	Poisson regression.	Thematic analysis. Comparing themes between high-adherence and low-adherence groups.

### 2.4 Measures

#### 2.4.1 Outcomes

The primary outcome variables include uptake and protocol adherence. Uptake, coded 1 or 0, refers to whether a participant registered an account in the application or not. 1267 participants took part in the study of which 306 participants registered in the app, representing an uptake rate of 24.2 %.

Protocol adherence is operationalized as the number of days on which a participant completed an intervention, expressed as a count variable coded 0–28. Participants completed the intervention on average 10.9 (*SD* = 8.52) days, with a skewness of 0.48 and kurtosis of 2.04. There was a large variance in adherence among participants, with participants completing anywhere between 0 and 28 days of the intervention.

#### 2.4.2 Predictors

Predictors consisted of demographic data including age, gender, occupation, and work-experience. Predictors also included measures of app engagement, burnout, stress, depression, anxiety, and PTSD.

**Burnout** was measured using the Oldenburg Burnout Inventory (baseline α = 0.88, pre-intervention α = 0.88), an instrument designed to measure exhaustion and disengagement from work (Halbesleben and Demerouti, 2005). This study used a Swedish translation with a subset of 7 items (Peterson et al., 2011). Items (e.g. “after work I often feel tired and exhausted”) are scored on a 4-point ordered categories scale (1 = Not at all, 4 = Exactly).

**Stress** was measured using the Perceived Stress Scale (α = 0.86, α = 0.89), a 10-item instrument designed to measure “the degree to which situations in one's life are appraised as stressful” (Cohen et al., 1983). This study uses Swedish translation (M. Nordin and Nordin, 2013). Items (e.g. “how often have you been able to control irritations in your life) are scored on a 5-point ordered categories scale (1 = Never, 5 = Very often).

**Depression** was measured using the PHQ-2 (α = 0.79, α = 0.78), a two-item screening tool designed to detect depression and anhedonia (Kroenke et al., 2003). This study uses a Swedish translation (S. Nordin et al., 2013). Items (e.g. “feeling down, depressed, or hopeless”) were scored on a 1–4 ordered categories scale (1 = Not at all, 4 = Nearly every day).

**Anxiety** was measured using the GAD-7 questionnaire, a 7-item instrument designed to assess generalized anxiety disorder (α = 0.89, α = 0.88) (Spitzer et al., 2006). This study uses a Swedish translation. Items (e.g. “Feeling nervous, anxious, or on edge”) were scored on a 1–4 ordered categories scale (1 = Not at all, 4 = Nearly every day).

**Post-traumatic stress** was measured using the PCL-5 (α = 0.82, α = 0.78), one of the most widely used self-reported measures of PTSD (Blevins et al., 2015). This study uses a Swedish translation with a subset of three items (Sveen et al., 2016). Items (e.g. “how much were you been bothered by: “Repeated, disturbing, and unwanted memories of the stressful experience?”) are rated on a 5-point ordered categories scale (0 = Not at all, 4 = Extremely).

**App engagement** was measured using the App Engagement Scale (α = 0.88), a 7-item questionnaire designed to measure engagement with mobile applications (Bakker and Rickard, 2018), translated into Swedish. Items (e.g. “I enjoyed using the app”) are scored on a 1–5 ordered categories scale (1 = Not at all, 5 = Fully agree). This measure was only included in the post-intervention measure (*M* = 3.44, *SD* = 0.78).

### 2.5 Data analysis

#### 2.5.1 Quantitative

All data analyses were conducted in R using the glm function (R Core Team, 2022). Complete analysis code and an anonymized subset of the data is available through an Open Science Framework repository (https://osf.io/ejh3f/).

To answer the first research question, a binomial logistic regression was conducted with uptake as outcome. All predictor variables from baseline measure were included.

To answer the second research question, a Poisson regression model was estimated since the outcome measure adherence is expressed as a count variable (Coxe et al., 2009). All predictor variables from pre-intervention measure were included. Additionally, a Spearman rank correlation was conducted between app engagement rating and adherence.

#### 2.5.2 Qualitative

Qualitative data analysis largely followed the steps suggested by Braun and Clarke (2006). Initially, the first author transcribed and read all interviews to get familiar with the text and form initial ideas. Subsequently, a subset of five interviews were selected to generate a coding scheme. A deductive approach was chosen in which the four components of the UTAUT framework formed initial codes – performance expectancy, effort expectancy, facilitating conditions, and social influences. The five interviews were coded according to these codes by reading the interviews thoroughly and fitting relevant passages to a corresponding code.

After coding the interviews, two codes were added to the UTAUT framework since these topics emerged as common topics in the data - *habit* and *intention*. Habit is a relevant category taken from UTAUT2, an extension of the UTAUT framework (Tamilmani et al., 2021). Intention is a key construct in the theory of planned behavior which has been integrated into multiple models of technology acceptance and engagement (Ajzen, 2020). The iterative approach of modifying the coding scheme to better capture interview content is a recommended strategy and common in the literature (Bixter et al., 2019; Goldsmith, 2021).

After generating these codes, two additional members of the research group coded the same five interviews according to this coding scheme. Inter-rater reliability with all three coders was measured using Fleiss´ Kappa (*k* = 0.69, *p* < 0.005) and was considered “good”, however, codes *facilitating conditions* and *social influence* did not have satisfactory inter-rater reliability (Fleiss et al., 2013). These codes also represented a small fraction of all codes (<10 %) and were thus removed after discussion with all coders. The adapted coding scheme had “very good” inter-rater reliability (*k* = 0.83, *p* < 0.005) according to the classification by Fleiss et al. (2013) and was chosen as a final coding scheme.

Compared with the original UTAUT framework, the final coding scheme replaced codes *facilitating conditions* and *social influence* with *habit* and *intention*. The final scheme included the following codes: (1) Performance expectancy - The degree to which an individual believes that using the system will help him or her to attain gains in health and well-being. (2) Effort expectancy - The degree to which an individual perceives the system to be easy and effortless to use. (3) Habit - The degree to which habitual behaviors influence use of the system. (4) Intention – The degree to which an individual has an intention to use the system.

The first author of the paper coded the rest of the interviews according to these codes, and subsequently searched for themes within each code. This part of the analysis employed an inductive approach, looking for common ideas and grouping together passages with similar sentiments. Importantly, this analysis was conducted separately within each user group (high-adherence, low-adherence, and no-uptake) so that themes could be compared between the groups. Lastly, relevant and compelling extracts from the interviews were selected in order to present and discuss in the article presented in Tables 5 and 6.

## 3 Results

### 3.1 Quantitative

#### 3.1.1 Uptake

Binomial logistic regression indicates that occupation was the only predictor affecting uptake of the intervention. Compared with assistant nurses, nurses (*OR* = 1.93, *p* = 0.04), physicians (*OR* = 3.46, *p* = 0.002), administrative staff (*OR* = 3.93, *p* < 0.001), and other occupations (*OR* = 3.47, *p* < 0.001) were more likely to start the intervention. Post-hoc analysis comparing estimated marginal means also indicates that other occupations (*OR* = 1.79, *p* = 0.04) were more likely to start the intervention compared with nurses. Table 3 shows the complete results from this analysis.

**Table 3:** Binomial logistic regression predicting uptake.

Variable	Estimate	*SE*	*OR*	95 % CI	*p*
Age	−0.01	0.01	0.99	[0.97, 1.00]	0.11
Work experience					
6–10 years	0.00	0.25	1.00	[0.62, 1.62]	1.00
11+ years	0.37	0.24	1.44	[0.90, 2,34]	0.13
Women	0.19	0.21	1.21	[0.81, 1.83]	0.35
Occupation					
Nurse	0.66	0.31	1.93	[1.05, 3.55]	0.04 *
Physician	1.24	0.34	3.46	[1.78, 6.74]	< 0.001 ***
Admin Staff	1.37	0.35	3.93	[2.00, 7.75]	< 0.001 ***
Other occupation	1.24	0.31	3.47	[1.89, 6.37]	< 0.001 ***
Depression	0.04	0.13	1.04	[0.80, 1.35]	0.78
Stress	−0.15	0.19	0.86	[0.59, 1.24]	0.41
Anxiety	0.27	0.22	1.31	[0.85, 2.01]	0.22
Ptsd	−0.08	0.11	0.92	[0.74, 1.14]	0.44
Burnout	0.16	0.17	1.17	[0.84, 1.64]	0.35

*Note:* Work experience, gender, and occupation have several levels. The reference level for Work experience is 0–5 years. The reference level for gender is men. The reference level for Occupation is assistant nurse.

#### 3.1.2 Protocol adherence

A poisson regression model indicated overdispersion (dispersion ratio = 4.05, *p* < 0.001), so a quasipoisson regression model was used to better fit the data (Ver Hoef and Boveng, 2007) The full results of this analysis are presented in Table 4.

**Table 4:** Quasipoisson regression predicting protocol adherence.

Variable	Estimate	Incidence rate ratio	*SE*	*t*	*p*
Age	0.00	1.00	0.01	0.43	0.67
Work experience					
6–10 years	0.13	1.14	0.16	0.84	0.40
11+ years	0.21	1.24	0.16	1.32	0.19
Women	0.18	1.19	0.14	1.22	0.23
Occupation					
Nurse	0.09	1.10	0.24	0.40	0.69
Physician	0.31	1.37	0.25	1.26	0.21
Admin Staff	0.17	1.18	0.25	0.68	0.50
Other occupation	0.18	1.19	0.24	0.75	0.45
Depression	0.02	1.02	0.09	0.25	0.80
Stress	0.01	1.01	0.12	0.10	0.92
Anxiety	−0.06	0.94	0.14	−0.44	0.66
Ptsd	0.00	1.00	0.07	0.06	0.96
Burnout	0.00	1.01	0.11	0.05	0.96

*Note.* Work experience, gender, and occupation have several levels. The reference level for Work experience is 0–5 years. The reference level for gender is men. The reference level for Occupation is assistant nurse.

Spearman rank correlation indicated a significant, positive relationship between app engagement rating and adherence (*r* = 0.23, *p* = 0.01).

### 3.2 Qualitative

The most common themes for each code are outlined in the following section. Table 5, Table 6 provide illustrative quotes for each theme and integrates quantitative and qualitative data to provide an overview of how themes contrast between participants of different engagement levels.

**Table 5:** Integrates qualitative and quantitative data by presenting and contrasting themes from users of different engagement levels. Groups are based on quantitative measures of engagement while themes are based on qualitative data analysis. Shows codes Effort expectancy and Intention.

High adherence	Low adherence	No uptake
Effort expectancy		
**Suitable amount of time and effort:** “*It was very simple and quick to use. So I don't think it was a large time commitment*” **Technical complications:***“The app was a hassle at times”*	**Too much time and effort:***“For me it was too often”* **Technical complications:***“Some technical issues as is the case sometimes”*	**Too much time and effort:***“Too much time commitment from my side to want to do it”* **Technical complications: *“****I had issues logging in and didn't have the time to fix it.”*

Intention		
***Sense of importance:** “I felt I was contributing to something important”* ***Sense of obligation:** “Since I had agreed to take part, it was natural for me to answer as often as possible”* ***Motivation:** “You need to be motivated to use it every day … an awareness about how you feel and a desire to change your health”*	**Sense of importance: “***I wanted to contribute to research and that made me feel engaged”* **Motivation***“In order to do it you need engagement to put aside time. And then I forgot and did not prioritize. That's why I didn't do it every day”*	***No sense of importance:** “Maybe if I could relate to being stressed and worried about these topics it would be different”* **Not enough sense of obligation:***“I did want to take part, some sort of civic duty you could say”*

**Table 6:** Integrates qualitative and quantitative data by presenting and contrasting themes from users of different engagement levels. Groups are based on quantitative measures of engagement while themes are based on qualitative data analysis. Shows codes Habit and Performance Expectancy.

High adherence	Low adherence
Habit	
**Daily format supports habit formation:***“I thought it was great to do it once daily, then you build a habit after 3–4 days. Then it's really easy. Had it been every other day I would've forgot it exists.”* ***Notification helps habit:** “Notification would help me remember sometimes”*	**Habit formation:***“It's probably on me. I should've prioritized to make it a routine to always do it”* ***Notification helps habit:** “It was my family situation and … having the reminder notification. That's what affected whether I would remember or not”*

Performance expectancy	
**Benefit of mood reflection***“I found it interesting to consider my mood every day, because usually I am bad at this. So I found it insightful to notice how I'm actually feeling.”* **Good prompts:***“I thought the prompts were good. All of them were useful”* **Feedback would be good:***“some sort of feedback or graph over your responses or how you're doing”*	**Benefit of mood reflection:***“So it was a good tool to help me measure my mood, or at least think about how I'm feeling.”* **Daily format not beneficial:***“Not every day but at the end of the week … that would have given more”* **Feedback would be good:***“To receive statistics which provide an easy to understand overview”*

*Note*: No themes reported for no-uptake participant within codes Habit and Performance Expectancy because these did not apply for participants who did not use the application.

#### 3.2.1 Effort expectancy

Time and effort: A primary theme to influence engagement was the perception of invested time and effort. Most uptake participants thought using the intervention was an overall smooth and easy process which took a suitable amount of time. On the other hand, no-uptake participants as well as some low-adherence participants often experienced that using the intervention was too large a commitment.

Technical complications: Participants in all groups experienced technical complications which were a source of frustration. For no-uptake participants, they sometimes had issues registering an account in the application which would then be a determining factor in them not starting the intervention. Other issues included being unable to access questionnaires, not receiving reminder notifications, and overall bugs making the application frustrating to use.

#### 3.2.2 Intention

Sense of importance: Feeling that this kind of mHealth intervention is important strongly influenced engagement. Uptake participants often reported that using the application felt important to them and that they were engaging in something meaningful. No-uptake participants, on the other hand, did not share this sense of meaning and did perceive the intervention to be important.

Sense of obligation: Feeling that one was obligated to use the intervention also influenced engagement. Uptake participants often mentioned they experienced a sense of obligation which would contribute to their engagement - once they had agreed to be part of the project they felt obliged to see it through, a tendency especially pronounced in high-adherence participants. No-uptake participants did not experience this obligation as strongly. Even in cases where they did feel a sense of obligation, it was not sufficient to start using the application.

Motivation: Being motivated to use the intervention was another contributing factor influencing engagement. High-adherence participants frequently reported that it requires motivation to consistently use the application. Low-adherence participants, however, did not feel the same motivation and would thus not prioritize or remember to do the intervention.

#### 3.2.3 Performance expectancy

Benefit of content: Most participants commented that the intervention content promoted beneficial personal reflection and inspired them to make positive change. The questions and prompts allowed users to think about their emotions and behaviors in productive ways, initiating an inner process that was valued by them. For instance, participants reported becoming more aware of emotional patterns and motivated to make changes in their behavior.

Lack of feedback: Many participants reported that they experienced a lack of feedback in the app and would have liked some functionality to easily track their own responses. For instance, this could have been a graph showing their reported values over the course of the intervention.

Daily format not beneficial: Some low-adherence participants did not appreciate the daily format, thinking it was too frequent to have an optimal effect. Rather, they thought that a weekly format would have been more beneficial. More time in between interactions would minimize the risk of making the intervention repetitive and instead encourage users to have fewer but more valuable interactions.

#### 3.2.4 Habit

Habit formation: The ability of participants to form a daily habit of using the intervention was a contributing factor to engagement. High-adherence participants often mentioned that the daily format was highly conducive to forming a habit, commenting that if the interactions were less frequent it would be more difficult to remember to do the intervention. Low-adherence participants often struggled more with forming this habit, not making a routine out of doing the intervention.

Reminder notification: Most participants commented that reminder notifications would greatly help in remembering to do use the application, some saying it was primary reason for remembering to do the intervention. Due to technical issues many participants did not receive reminder notifications which negatively affected their engagement.

## 4 Discussion

### 4.1 Key results

Results from the study indicate several factors influencing uptake and adherence, providing important insights for how to increase engagement with organizational mHealth interventions. Some factors, such as app engagement and perceived time commitment, are related to the structure of the intervention and can be influenced by adapting the app design and intervention format. Other factors which impact user engagement, such as the user's occupation and intention, are largely unrelated to the mHealth application and are more likely to be addressed by implementation strategies in the workplace.

The mixed method design allowed us to uncover the specific reasons participants had for engaging with the app to the extent they did. By dividing participants based on quantitative measures of engagement and contrasting interview data between these groups, qualitative differences between these groups emerged. Conclusions regarding how to improve the intervention design for low-engagement users would not have been possible without the mixed methods study design.

1) *Short and consistent interactions increase engagement through perceived ease of use and supporting habit formation.*

A key take-away is that app engagement rating was significantly correlated with adherence - participants who rated the app more highly also tended to use it more often. The app engagement scale primarily measures aspects of engagement related to the design of the application, for instance asking participants if they found the application motivating, easy, and enjoyable to use. This result indicates that a positive experience of using the app is linked with higher adherence rates and that, fortunately, spending resources on designing engaging and usable applications may yield beneficial results (Bakker and Rickard, 2018; Stoll et al., 2017).

Of course, it is important to uncover what specific aspects of the application design contribute to increased engagement. According to the qualitative analysis, a critical factor is the perceived amount of time and effort involved with using the application. More engaged participants consistently thought the daily time commitment was reasonable, compared with low-adherence and no-uptake participants who often found it too demanding. Technical issues which increased the expenditure of time and effort frequently emerged as a factor which negatively affected engagement. In the literature, perceived ease of use is a well-known factor predicting technology acceptance and our results are consistent with this finding (Marangunić and Granić, 2015).

Experiencing the intervention as too time-consuming and effortful proved to be especially detrimental in the uptake process. No-uptake participants reported that once they ran into a technical issue or experienced confusion while registering an account in the application they gave up registration, even though they had an intention to start using the application. This points to the importance of having an uptake process which is as effortless as possible, ensuring that everyone with the intention of starting the intervention does not face initial resistance.

Ensuring interactions with the application are sufficiently effortless is also important with regard to helping users form a habit of using the intervention. Habit formation benefits from small and incremental behavior change with consistent repetition (Lally and Gardner, 2013; Sanders et al., 2021) – thus, an intervention format with brief and frequent interactions may be advantageous for increasing engagement. Indeed, high adherence users repeatedly reported that the daily short-form structure was conducive to forming a habit which contributed positively to adherence.

2) *Workplace encouragement may improve engagement through increasing perceived benefit and use intention among employees*.

Finding the application engaging only explains part of the variance with regard to user engagement (*r* = 0.23 with adherence), and other factors also impact engagement with the intervention. A primary factor influencing both uptake and adherence is intention – participants who felt compelled to use the intervention were more likely to have higher engagement. This intention was commonly related to feeling that the intervention was somehow important, being motivated to use it, or experiencing a sense of obligation to complete the intervention.

That intention emerged as a primary factor influencing engagement provides an avenue where stakeholders - researchers, employers, health-care professionals - can focus resources to improve engagement. Kim et al. (2013) suggest that utilitarian and social motivations affect user engagement intention. Our results are partly consistent with these findings, indicating that perceiving the intervention as beneficial was an important factor contributing to increased engagement. Raising awareness regarding the intervention's utility may be a way of further increasing perceived benefit and thus engagement.

We did not, however, find evidence of social motivations contributing to engagement. Themes related to the construct “social influence” of the UTAUT framework did not emerge in the qualitative data, suggesting that this construct was either unimportant to participants or that social motivations were absent during the intervention. In our intervention, there was no strategy in place to foster a social atmosphere encouraging the use of the intervention. Given that social influence has previously been found to be a significant driver of engagement, this motivation may have been underutilized in our intervention affecting engagement negatively (Zhou et al., 2019).

Leveraging utilitarian and social motivations in the workplace are thus potential avenues for enhancing engagement with organizational interventions. Management could, for instance, communicate the value of the intervention to employees, emphasizing its potential benefits (von Thiele Schwarz et al., 2021). Additionally, it is important to cultivate a social atmosphere which is supportive of intervention use. This study did not explore how to leverage these motivations in the workplace, a point which is expanded on in the Limitations section.

3) *Targeting specific occupational groups may be important to reach all employees.*

The findings suggest varying levels of engagement between different occupational groups. Assistant nurses were less likely to initiate the intervention compared with most other health-care occupations, something which could be related to education and technological literacy. Previous work has indicated that that having more educational qualifications is linked with higher engagement levels (Garnett et al., 2018; Szinay et al., 2020). Given that assistant nurses typically have fewer educational qualifications than other occupations in the health-care sector this is a possible barrier to engagement. Technological literacy, associated with educational level, is also a driver of mHealth use and could similarly affect engagement among assistant nurses (Yardley et al., 2016; van Deursen et al., 2011).

Language barriers may also affect uptake among assistant nurses. In Sweden, it has been observed that a substantial proportion of assistant nurses may not have adequate Swedish language skills (Statens Offentliga Utredningar, 2019:20). Since the intervention was offered in Swedish, it may be of less interest to users who are less proficient in the Swedish language. Providing the intervention in a language suitable for users may be a viable strategy for increasing uptake (Povey et al., 2016).

Nurses were also less likely to start the intervention compared with other occupations. This is an occupational group which during the Covid-19 pandemic and the time of data collection were offered extensive opportunities to partake in different support interventions (Maben and Bridges, 2020). For this reason, they may not experience the same need for additional mHealth support as occupational groups who were not offered other support initiatives to the same extent.

Considering that different occupations within the same organization show different levels of engagement it may be beneficial with implementation strategies that motivate occupational groups less likely to use an intervention. Identifying which occupations are less likely to engage with the intervention and implementing appropriate strategies to encourage engagement among these employees may be a viable strategy for increasing overall engagement.

### 4.2 Limitations

Data were collected from health-care workers during the Covid-19 pandemic which placed extreme demands on employees during the time of the intervention. For this reason, it may be difficult to generalize claims regarding engagement and how a similar intervention would be received during more regular working conditions. At the same time, since the intervention was tested during such a difficult time and still had promising uptake and adherence rates, this could be a positive sign that even in challenging conditions it is possible to engage people to use mHealth interventions (Bartels et al., 2020).

Other limitations include technical issues in the application which negatively affected the quality of the intervention and data collection. The software used to conduct the intervention was developed in a limited amount of time given the need to quickly implement an intervention and data collection tool during the Covid-19 pandemic. Given these circumstances, there was insufficient time for a thorough development and testing process, leading to technical complications in the application. Results may be affected by these issues that are unlikely to generalize to real-world scenarios with properly developed digital interventions.

Lastly, qualitative data analysis was conducted largely by the first author of the paper which may be a source of bias. To mitigate this, a subset of interviews was analyzed by several members of the research group and the coding strategy was adapted until a satisfactory inter-rater reliability was achieved. Even so, having a single person conduct most of the qualitative analysis increases the risk of bias and missing important themes in the interview data.

### 4.3 Future directions

An important finding from this study is the influence of user intention on engagement in an organizational context, specifically its effect on uptake and adherence. Future research could explore this relationship more in-depth and investigate how to positively influence intention among employees. Social motivations - such as management encouragement and a supportive workplace culture around the intervention - could be key pathways for increasing intention. However, these factors were largely underexplored in this study and it is not clear how to effectively create a social workplace dynamic supporting engagement.

An explorative qualitative study investigating employee needs regarding social motivations for mHealth interventions may be a fruitful first step in this direction. The study could, for instance, employ interviews with a diverse set of employees to generate ideas regarding what would be effective social motivation. Additionally, focus groups including both employees and management could prove important to receive multiple perspectives on this issue and foster meaningful discussions. These insights may then be used when implementing future interventions and subsequently evaluate results from these.

Another avenue for future research involves identifying which occupational groups within organizations are less likely to engage with workplace mHealth interventions and developing strategies for engaging these groups. Our data indicate that assistant nurses and nurses may be such groups, however, more knowledge is needed to identify low-engagement occupations in other sectors. Review studies, for example, could potentially discover trends that predict which types of occupations are likely to exhibit lower engagement. Resources and strategies for increasing engagement can then be targeted at these groups.

## 5 Conclusions

Results from this study indicate several strategies for improving engagement with organizational mHealth interventions. Firstly, short and consistent interactions are suggested for not overburdening users and also support building a habit of using the intervention. Another impactful predictor of engagement is an intention to use the intervention. This could potentially be influenced by environmental workplace factors such as encouragement from management and the cultivation of a supportive social atmosphere around the intervention. More research is needed regarding how to effectively leverage these motivations in order to increase engagement among employees.

## Funding

The project was funded by a grant from 10.13039/501100004359Vetenskapsrådet, dnr. 2020-05800.

## CRediT authorship contribution statement

Leo Kowalski has contributed to conceptualization, data curation, data collection, formal analysis, methodology, project administration, and writing. Anna Finnes has contributed to conceptualization, supervision, funding acquisition, reviewing, and editing. Sabine Koch has contributed to supervision, methodology, reviewing, and editing. Aleksandra Bujacz has contributed to conceptualization, data analysis, project administration, funding acquisition, methodology, supervision, reviewing, and editing.

## Declaration of competing interest

The authors declare that they have no known competing financial interests or personal relationships that could have appeared to influence the work reported in this paper.

## Appendix A Supplementary data

Appendix Interview guide for semi-structured interviews. Appendix
