# Supplementary material for: User engagement with organizational mHealth stress management intervention – A mixed methods study
Source: Internet Interv. 2024 Jan 2;35:100704. doi: 10.1016/j.invent.2023.100704 (PMC10806331; doi:10.1016/j.invent.2023.100704)
Supplement: Appendix — Interview guide for semi-structured interviews. [file mmc1.docx]

**Appendix – Interview guide for semi-structured interviews**

Note: This is merely a guide which structured the semi-structured interviews. The questions were not necessarily asked in this particular order or exact wording. The interviews were more of a conversational and open format, in which participants could talk about topics outside these questions in case those topics came up and were deemed relevant for the study. The questions below were used more as a basis to ensure the interviewer remembered to cover the most important aspects of the qualitative data collection. The questions were slightly different based on the engagement level of the participant.

High–adherence participant answered more than 14 times

1. **Did the intervention give you anything? What value did it have? How was it to use DIARY?**
   1. Was it valuable with the reflection and prompts?
   2. What reflections/prompts were better or worse?
   3. What reflections/prompts do you feel were missing?
2. **What were the reasons behind engaging with the intervention?**
   1. Were you engaged before or was it due to the intervention format/app?
   2. What made you keep up engagement?
   3. Was there something which negatively affected engagement?
   4. What would positively influence engagement?
3. **How did the daily format feel?**
   1. Too often, too rarely?
   2. Was it too much/little work? Would you have preferred another structure?
   3. In what ways could one decrease the effort?
4. **In what ways could this kind of application be used?**
   1. Would you like to have this in the future?
   2. In what ways do you think this kind of intervention could and should be used?
   3. For whom and when is this kind of intervention appropriate?
   4. What additional functionalities would be useful?

Low-adherence participant – Answered less than 14 times

1. **How was it to use the intervention? Has it given you anything? What value did it have?**
   1. Was it valuable with the reflection and prompts?
   2. What reflections/prompts were better or worse?
   3. What reflections/prompts do you feel were missing?
2. **What made you use the intervention to the extent that you did?**
   1. Was it due to engagement?
   2. Where you engaged before starting or was it because of the format/application?
   3. What affected engagement negatively?
   4. What factors would positively influence engagement?
   5. Did you experience any specific barrier to engagement?
3. **How was the daily structure?**
   1. How did you feel about the daily structure? Too often, too rarely?
   2. Was it too much/little work? Would you prefer another structure?
   3. In what ways can one decrease the effort?
   4. How could we encourage people to engage more?
4. **In what ways could this kind of application be used?**
   1. Would you like to have this in the future?
   2. In what ways do you think this kind of intervention could and should be used?
   3. For whom and when is this kind of intervention appropriate?
   4. What additional functionalities would be useful?

No-uptake

1. **Why did you choose not to download the application? What was the obstacle?**
   1. Did not understand the extent of the study protocol?
   2. Did you feel it was effortful with the intervention format and/or technology?
   3. What could have helped you to download the app and follow the study protocol?
   4. What felt challenging?
   5. Could we change anything to lower the threshold?
   6. What would have made you want to use the application?
